# Economic models for prevention: making a system work for patients

**DOI:** 10.1186/1472-6831-15-S1-S11

**Published:** 2015-09-15

**Authors:** Michael Helgeson

**Affiliations:** 1 Apple Tree Dental, Corporate Office, 8960 Springbrook Drive, Suite 150 Minneapolis, MN 55433, USA

## Abstract

The purpose of this article is to describe alternative means of providing patient centered, preventive based, services using an alternative non-profit, economic model. Hard to reach, vulnerable groups, including children, adults and elders, often have difficulties accessing traditional dental services for a number of reasons, including economic barriers. By partnering with community organizations that serve these groups, collaborative services and new opportunities for access are provided.

The concept of a dental home is well accepted as a means of providing care, and, for these groups, provision of such services within community settings provides a sustainable means of delivery. Dental homes provided through community partnerships can deliver evidence based dental care, focused on a preventive model to achieve and maintain oral health.

By using a non-profit model, the entire dental team is provided with incentives to deliver measurable quality improvements in care, rather than a more traditional focus on volume of activity alone. Examples are provided that demonstrate how integrated oral health services can deliver improved health outcomes with the potential to reduce total costs while improving quality.

## Introduction

There are various systems to remunerate dentists and each of these are based within the context of the national healthcare system within which they operate. For example, there are fee for item services that pay for individual elements of work, and, at the other extreme there are capitation based services that aim to pay a single fee to secure a patient, or population's, oral health. Both approaches have been criticized - the fee per item incentive can lead to over treatment while capitation schemes are sometimes seen as rewarding supervised neglect. Insurance and national based payers are exploring hybrid systems that aim to blend these approaches [1]. Irrespective of the payment system, the challenge of ensuring that services are available to high needs, and difficult to reach groups, remains [2][3].

The changing demographic in the US needs to be considered alongside this challenge. The U.S. population is transitioning from one dominated by younger individuals to a population more evenly spread across the age spectrum [4]. The baby boomer generation is now reaching the age of sixty-five and is largely responsible for the increase proportion of elders within the overall population [4]. This increase in elders is mirrored by an increase in overall oral health and hence this group will be maintaining their natural dentition into old age, and the proportion of older adults with complete dentures is decreasing [5]. Older adults are just one group that will challenge the provision of dental services in the future and contribute to the complexity of care required for those not traditionally served by primary care dental practices [6].

This paper describes an approach to a preventive focused care system using a non-profit delivery model for such groups that aims not only to provide access but also genuine improvements in oral care.

## Aims of non-profit oral health care systems

The “triple” aim of US health care systems are improving the experience of care and improving the health of populations while reducing the total cost of care [7]. Such an aim is not unique to the US, but is shared by most other Western health care systems where very similar goals have been advocated. The US, however, spends more on health care than any other developed country [8], and yet, the outcomes of this high cost system are some of the poorest outcomes for population based health care measures [9]. Paradoxically, studies have shown an *inverse* relationship between spending and outcomes [10] and the same is observed in oral health [11].

Typically dental services are focused on “one patient at a time” and as a largely surgical discipline, seek to treat disease in a mechanistic fashion, often ignoring the underlying causes of the disease. Throughout these proceedings authors have advocated for the holistic assessment of patients, and the use of preventive, rather than surgical, approaches to care. In order for this approach to realize real health improvements the individual health behaviors, genetic factors, and environmental factors of the population must be considered. These factors account for about 90% of healthy outcomes, whereas healthcare services account for only 10% [12].

It is therefore key that any new service, especially a non-profit provider, cannot be purely focused on the delivery of traditional dentistry, but must engage with the target population on various levels, promoting health changes in the environment and facilitating change. Indeed, given the 90-10 split in health outcomes, a small change produced in this area can result in greater benefits than those secured by the provision of traditional dental care.

### The traditional private practice system is primarily a reactive system

In the United States, most dental care is provided in either private for-profit dental offices or in public safety-net dental clinics called Community Health Centers. Both settings can be described as surgical suites and they react after patients have identified their need for services and have called to request an appointment for dental care. The success of these traditional approaches depends on high levels of dental literacy, such as the knowledge that regular preventive visits are needed in the absence of any symptoms, and also on appropriate financial resources. Approximately 70% of the U.S. population has these characteristics and interacts successfully with traditional dental providers [13]. This segment of the public enjoys very good oral health status, low rates of new mouth infections and high rates of restored and functional teeth [14].

However, “hard to reach groups” including older adults, people with physical and learning disabilities, those in institutional care or those with limited access to financial resources cannot act as proactive patients by themselves, and require advocacy on the part of carers or family members. The concept of a “proactive responsible party” is outlined in Table 1. As can be seen, this is a fairly comprehensive list and few individuals will be able to fulfill one or more of these requirements. Medicaid provides little or no coverage for adults even in those states where it is provided. Even when patients have sufficient resources to access care on a private basis, there are still significant barriers to access; often in relation to physical access or concerns from the dental provider over complex medical conditions or cognitive impairment.

**Table 1:** Key items on the Proactive Responsible Party's Dental Checklist.

Proactive Responsible Party's Dental Checklist
□ I'm motivated to take the lead on behalf of an aging or disabled vulnerable adult. Obtaining routine dental care is a high priority to me, whether or not my ward has obvious dental problems.
□ I am willing and able to find a dental office near myself and also near the nursing facility or group home that meets these requirements:
□ The dental office accepts new Medicaid patients
□ The dentist is skilled at treating medically, behaviorally, and dentally complex vulnerable adults
□ The parking lot, building and dental office chairs are all wheelchair accessible and the dentist's staff are skilled in safe patient handling
□ My employer is willing to approve time off so that I can accompany a vulnerable adult for dental appointments. This is in addition to approving time off for my own dental appointments several times each year, as needed.
□ I have my own personal funds to pay for my own automobile or for public transportation so that I can travel from home or work, to a group home or nursing facility and also travel to a nearby dental office, and of course, back again, several times each year, as needed.
□ I have been formally designated as the “responsible party” and I have the clear authority to act on behalf of the patient for medical care and financial decision-making. I feel comfortable carrying out this role, and I understand that it includes being responsible for regular dental care.
□ I will direct nursing facility staff members to coordinate and schedule medical transportation services for dental visits as needed.
□ I will authorize the nursing facility staff to release medical, nursing and financial information as requested by the dental practice.
□ I will assure the dental practice that a nursing staff member will accompany the vulnerable adult during transportation to and from each periodic dental appointment, and will assist during dental care if necessary.
□ I will participate actively during the dental evaluations on behalf of the vulnerable adult, considering all treatment options and the limitations of Medicaid dental coverage, and I will authorize appropriate and necessary dental care, even if I have to find an alternative way to pay for services not covered by Medicaid.
□ Because regular dental care is so important to me, I am willing to repeat this process twice a year for checkups, plus one or two additional visits for treatment that may be necessary.

Consideration should also be given to the costs incurred by the responsible party - these may be actual financial costs - for example time off work, travel, child care arrangements (and can add up to several hundred dollars for each visit, which may well be in excess of the dental care costs) but there will also be opportunity costs which should be considered as well. The provision of care in traditional settings can be unsettling for medically complex adults and children - a long transport period combined with unfamiliar surroundings can often trigger counter productive behaviors at the dental surgery further complicating, or in some cases, preventing, the delivery of care. Given these complexities it is perhaps not surprising that a recent nursing home survey found that only 13% of residents had a documented dental visit during their stay [15]. It is unlikely that this figure would be much different for those individuals living within the community with family provisioned care (by far the larger group).

### The development of proactive oral health care delivery systems

Apple Tree Dental (ATD) is used as an example of a non-profit, staff model, accountable and sustainable oral health care delivery system [16]. ATD proactively targets community organizations where high-risk, hard to reach (and thus underserved) populations live, work, go to school or receive other services. Such organizations include Head Start Centers, those serving adults with disabilities and facilities serving seniors and frail elders. It builds on the conceptual framework of a dental home [17] but expands and enhances the model with proactive and responsive systems for delivery of care.

Once identified a *proactive* program of tailored oral health interventions are developed and implemented in collaboration with the community partner. The focus of the interventions is to prevent mouth infections, deliver evidence based preventive care of the types articulated within the proceedings of this conference, and undertake surgical dentistry where appropriate to stabilize and sustain oral health. ATD's model is not a simple mobile dentistry service based on the provision of sporadic, intermittent, fragmented or urgent symptomatic care - rather it is designed to provide ongoing, sustainable year round access to care.

## The Apple Tree Dental (ATD) Model

ATD is an independent, nonprofit, staff model group practice that was established in 1985 with a commitment to serving nursing home residents in the Twin Cities of Minneapolis and St. Paul, Minnesota. ATD's mission is to improve the oral health of people with special access needs who face barriers to care. Since its founding, ATD has delivered more than one million dental visits and screenings and provided more than $175 million in oral care services.

ATD was inspired by the Mayo Clinic's approach to nonprofit group medical care provision. As such, ATD is led by a volunteer Board of Directors with expertise in areas such as health care administration and research, dentistry, public policy, nonprofit governance, and epidemiology. Apple Tree's paid professional staff has grown to more than 200 with an annual budget over $25 million. An interdisciplinary group practice provides a business model that builds a team with expertise in the many areas needed to design unique geriatric, pediatric, and adult dental programs that fully integrate with the work of teachers, nurses, physicians, and others. Leadership team members have expertise managing interdisciplinary teams working in a variety of settings, carrying out program and project evaluations, fundraising, finance and administration, implementing internal and external education programs, and promoting policy development and dental access legislation. The approach is also consistent with the Anderson Behavioral Model of Health Services - the three dynamics of predisposing factors, enabling factors and need are reflected in the model [18]. In terms of pre disposing factors the model targets communities with known high needs, yet suboptimal use of dental services. Sites chosen are those where these high risk, low access populations live, work, and go to school. In relation to enabling factors formal contracts with community organizations, such as skilled nursing facilities or schools, create a social support network that leverages access to insurance, care coordination, transportation, etc and creates an environment where oral health literacy is elevated. And finally, in relation to need the system of care, as supervised by the Dental Director role includes oral health education and in-the-mouth screening, assessment and referral of everyone at each setting. This identifies objective problems early, even when there was no perceived need or subjective complaint. It also promotes health literacy, and creates links to coverage options when necessary.

Key features of the ATD model include:

• Patient centered, tailored to serve people with special needs and other underserved populations

• Focused on prevention and early detection to keep people healthy

• Providing evidence based treatments that achieve sustainable health outcomes

• Geographically distributed, targeting places where high risk patients congregate

• Collaborative, linking dental professionals with doctors, nurses and other caregivers

• Telehealth enabled, speeding up diagnosis, care coordination and follow-up care

• Accountable dental homes, that provide year-round access to comprehensive care

## Developing collaborative practice

The delivery of on-site dental care requires a collaborative community approach - a tripartite arrangement between the dental service provider, the community organization and on-site oral healthcare teams. The process of this collaborative approach begins with meeting the leadership of the nursing home, school or Head Start Center and makes it clear, from the outset that the success of the program will be a joint effort. An on-site oral health program is developed and a written “oral health services agreement” establishes legal relationships between the parties articulating the roles and responsibilities of each organization, addresses applicable regulatory requirements, provides for health and financial information exchanges, addresses liability issues, discloses financial arrangements, and addresses other operational issues.

In this model ATD's role is to act as the Dental Director for the community organization - in much the same way as a nursing facility's Medical Director. Many organizations require that the Dental Director take responsibility for assuring access to oral health and dental care services is made available to everyone at the site without discrimination. Other responsibilities vary depending on the type of community organization. For example, Head Start Centers have federal dental examination requirements to meet, while nursing facilities have both federal and state licensure requirements regarding routine and emergency dental care policies and practices.

## Delivery of care

Using a hub and spoke system, the ATD model of care utilizes portable and mobile equipment to provide care and these have been developed and adapted over the 30 years of service provision. The equipment is delivered using specially designed trucks that can serve multiple sites each day via a carefully planned route. Drivers pick up and drop off one or more complete mobile offices at each location - finishing their deliveries by midnight. The following morning, dental care teams arrive, and within 15 minutes of set up, are able to provide care for one or more days before the mobile office is picked-up for use at another site.

In addition to using complete mobile dental offices as described above, ATD also employs collaborative hygienists who provide front-line oral health services using equipment they can transport in an automobile. Using this smaller, lightweight portable equipment makes it possible for them to attend several sites in a single day. Collaborative hygienists provide oral health services including patient and caregiver education, preventive services, daily mouth care planning, in-the-mouth assessments, and early intervention services that can be provided in the resident's own room.

## Can new models of care delivery achieve prevention in practice for hard to reach groups?

In addition to the triple aim in health care, the Institute of Medicine has defined six quality requirements for health care delivery systems [19]. These apply equally to dental services; by exploring each of these, we can demonstrate how non-traditional care systems can deliver high quality care with a focus on prevention:

### Safe

Safety is paramount in all services, and for those orientated to individuals with special needs, physical or mental disabilities or frail elders the avoidance of injuries, complications and trauma is especially important. Indeed, traditional dental service providers often raise safety concerns as a reason for failure to accept such patients. On-site care systems promote and engineer safety into their services through direct, face-to-face inter-professional interactions. The real time interaction between all of the professionals involved in a patient's care reduces communication errors, improves the management of patient behaviors, facilitates the management of drugs and medications, and improves physical safety using staff and equipment for wheelchair to dental chair transfers. The joint working approach also fosters a sense of interdisciplinary accountability for the care of patients further enhancing the safety element of such services.

### Effective

As has been articulated in the proceedings of this conference, the use of evidence based preventive strategies underpins a medical (preventive), as opposed to surgical (restorative) approach, to care delivery. Preventive systems, as described by Birch in this supplement, can be cost effective, and the use of clear governance and management systems in new models of care ensure that they are employed while reducing reliance on excessive or unnecessary treatments. Whether or not each patient and their personal caregiver is able to complete an effective regimen of daily mouth care continuously is dependent on an effective culture within the health care facility that supports, monitors, and rewards effective daily mouth care. On-site care systems are designed to promote institutional “culture change” within the residential facility. To reach the goal of healthy patients who are stable at each periodic dental evaluation, patient behaviors and the environment make a bigger difference than the technical dental/surgical procedures delivered. When patients are undergoing more complex dental treatments, such as removal and replacement of teeth with dentures, the impact of direct caregivers on the success and effectiveness of specific treatments cannot be underestimated. Whether or not a resident is able to adapt and function well with new dentures, for example, hinges as much on the skills and collaborative role of the Personal Care Assistant, Certified Nursing Assistant or family member as it does on the dentist and denture lab technicians who fabricated the prosthetic devices.

### Patient centered

New models of care delivery are responsible to individual patient needs, preferences and values, as well as considering the wider population approach. The interactions with the responsible party, the dental team and the wider inter-professional group help guide decision making to ensure that it is patient centered. The approach to the ethics of care is essential and the use of shared learning within inter-disciplinary teams adds real value to formal classroom and live mentoring. Culturally competent care, that recognizes and respects differences in patients is essential and the use of a flexible schedule removes the concept of a time limited dental appointment and replaces it with a patient orientated care experience. Patients and the dental team are present all day - and hence the time taken to treat an individual is not governed by a pre-determined appointment length, but rather by a patient's clinical and medical needs. For example, a patient with dementia may be agitated in the morning, and hence their care can simply rescheduled to a later afternoon appointment. In a traditional setting this may have resulted in failed dental visit.

### Timely

Dental services delivered on-site throughout the year can reduce potentially harmful delays in both diagnosis and treatment for high-risk patients. Through pro-active engagement with partners, the provision of oral health education and oral health assessment at admission virtually eliminates the need for symptomatic care. Screening enables timely care for residents and the introduction of preventive regimes further secures oral health. This approach enables a continuously tailored schedule of on-site care, using a range of providers, to ensure that preventive, diagnostic and restorative care is provided as necessary as well as ensuring that, when needed, urgent care is provided promptly.

### Efficient

Efficiency must be focused on securing optimal health outcomes, rather than simply delivering faster services without improving outcomes. An example from the personalized care section shows how this can work - by ensuring flexibility within the system, failed treatment sessions are reduced and hence the efficiency of the service is increased. The process of obtaining consent, especially time consuming in patients with learning disabilities or those who are cognitively impaired, is undertaken *prior to dental treatments* and hence improves the efficiency of treatment sessions. The shared accountability for oral health, with the patients, their representatives and staff, as well as the clinical dental team, establishes a healthy environment that supports health behaviors and hence the delivery of daily mouth care, reducing the treatment burden.

As previously described, the transportation of patients to traditional settings, is both time consuming and costly as well as potentially upsetting to patients. The zero travel time for onsite care avoids these costs and eliminates the “no-shows” seen in dental offices. The on-site dental team is able to provide care during the entire day with any spare appointment time at the end of each day filled with routine periodic examinations.

### Equitable

An essential element of the community collaborative practice model, embedded within a legal contract, enables the Dental Director to ensure equitable access to oral care. This is achieved through contract provisions including nondiscrimination clauses, and other provisions that establish standards of care that parallel medical, nursing and pharmacy standards. Health care facilities are committed to providing care that does not vary in quality because of personal characteristics such as gender, age, ethnicity, geographic location, and socioeconomic status. Without this type of formal relationship to ensure equitable access to care, vulnerable patients who are Medicaid recipients, people with behavioral challenges, and people without proactive responsible parties experience inequities in both access to care, and oral health outcomes. Such inequalities have been seen in dental programs, that refuse to serve Medicaid patients or those whose medical or behavioral conditions made them complex to treat.

By integrating oral health education, screening, prevention and referral into the “culture” of each community organization, and creating standard operating processes that begin at the time of admission (not waiting until a subjective complaint is raised) people who lack the knowledge to seek care are intercepted early and guided to the care they need.

## Conclusions

The conference proceedings have advocated for the introduction of prevention into dental practices. Examples have been provided demonstrating the evidence base for such therapies and Bridgman and McGrady suggest how this may be achieved within a general dental practice system. The introduction of prevention is as, if not more so, important for those in hard to reach and high-risk groups. The current funding models for mainstream dentistry continue to ignore access for these populations and therefore there is a clear role for alterative service providers to demonstrate innovation within the provision of care. The Apple Tree Dental model is provided here as example of how this can be achieved, operating within a context of the US as a non-profit dental model, bringing real patient benefits and larger population health gains.

## Competing interests

The author received funding from the Colgate Palmolive company to attend the Prevention in Practice Conference.

## Declarations

This article has been published as part of *BMC Health Services Research* Volume 15 Supplement 1, 2015: Proceedings of Prevention in practice - making it happen. The full contents of the supplement are available online at http://www.biomedcentral.com/bmchealthservres/supplements/15/S1. Publication charges for this supplement were funded by Colgate Palmolive.
